# Smallpox Eradication: African Origin, African Solutions, and Relevance for COVID-19†

**DOI:** 10.4269/ajtmh.20-1557

**Published:** 2021-01-18

**Authors:** Joel G. Breman

**Affiliations:** Fogarty International Center, National Institutes of Health, Bethesda, Maryland

## PREFACE

Thank you Annie (Rimoin) for those introductory comments. I can assure those tuned in that some of those stories are actually true. And, I am proof that there is no age discrimination in the ASTMH.

The Society has been my main professional home for five decades. This year has been like no other in our history—the COVID-19 pandemic has challenged us in every way. We have responded to the assaults on science through writing for our *Journal* and for other journals, joining like-minded societies with statements to the press and live interviews, and appealing directly to decision-makers.

Advocacy for global health research and control programs in poor countries has been led by our CEO Karen Goraleski. She, with our great staff and partners, has been performing miracles. Thanks team TropMed. And a big thanks to our board of directors, who set the standards high and are major advocates for what we do.

The biggest challenge has been to have this virtual meeting. I thank Dan Bausch and Stephanie Yanow, chair and associate chair of the Scientific Program Committee, and Lyn Maddox, vice president for meetings, for this feat, plus those understanding folks in Toronto whom we hope to see in the future.

Most dear to me is my family. My wife, Vicki—nurse, lawyer, chaplain, house manager, and fellow-traveler—has had good reason to serve me with divorce papers for frequent abandonment. She hasn’t...yet! My son, Matthew, and daughter, Johanna, and their spouses, Rachel and Tomer, are also stars with rich international histories and horizons. Thanks to them for constant support and guidance, and for their wisdom in raising kind, curious, and generous children of their own. May my grandchildren, Sebastian, Aviva, Leora, Eden, Arielle, and Felix get into good trouble on their own.

Now, for the main event. First, I would like all those who have worked on and supported the smallpox eradication program to stand—wherever you are. You are the heroes I honor today, who were celebrated yesterday in the smallpox symposium and mentioned by Dr. Tedros Adhanom Ghebreyesus, Director General of the World Health Organization, in his opening remarks.

**Figure:**
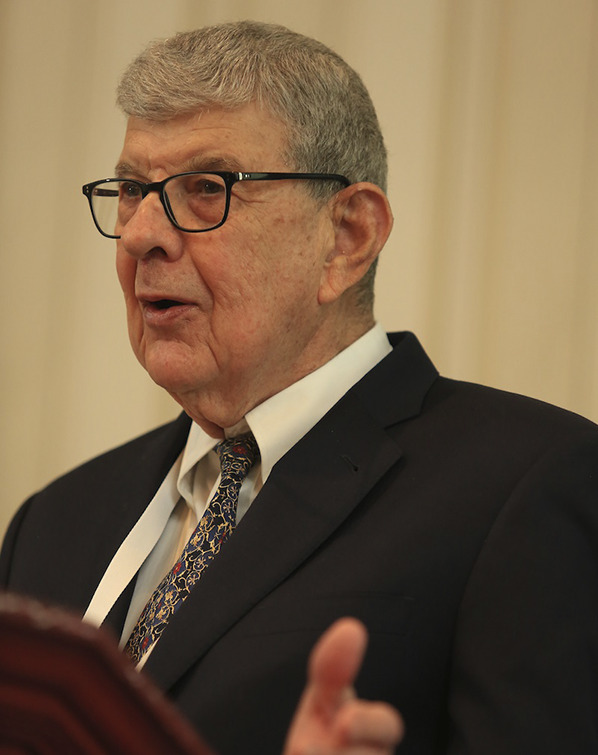
**2020 President Joel G. Breman, MD, DTPH, FASTMH**

## INTRODUCTION

2020 marks 40 years since the World Health Assembly accepted the 1979 conclusion of the Global Commission for the Certification of Smallpox Eradication that the dread disease had been eradicated.^1^ It is fitting that we reflect on the history and conduct of the eradication program, focusing on Africa. Not only is Africa where the first and last cases of the disease were recorded, but where some of the unique control and eradication strategies and tactics were discovered and deployed. The control of the COVID-19 pandemic will be dependent on many of the principles and practices used in smallpox eradication, another reason to examine our past.

## SMALLPOX IN AFRICA

**Figure 1.**
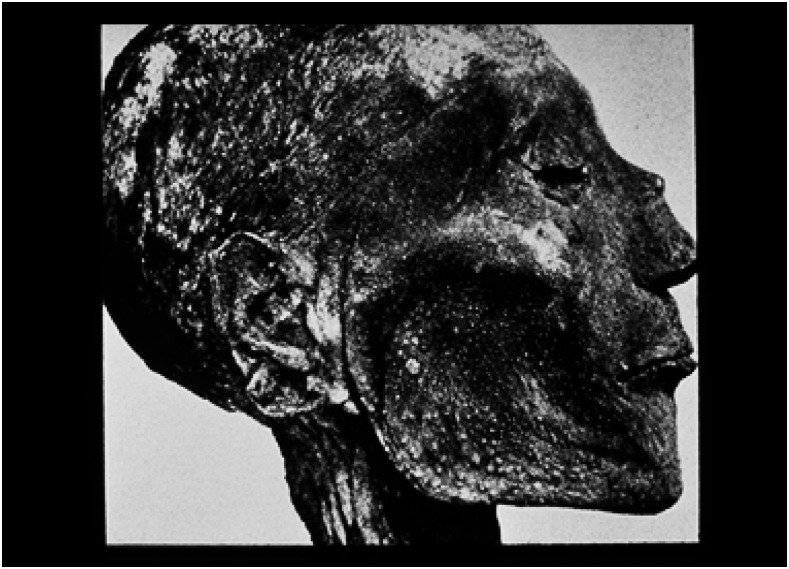
Ramses V, 1157 BCE. Source: WHO.

The Pharaoh Ramses V died in 1157 BCE. He has the dubious distinction of being the first known patient with smallpox, caused by variola virus. His mummified face had a set of pustules when the corpse was unwrapped and photographed in the early 1900s (Figure 1).^2^ Variola virus needs a critical number of humans to continue circulating, as there is no nonhuman reservoir of smallpox.^3^ The fertile crescent between the Tigris and Euphrates rivers was one of the most dense population centers in the world before the time of Christ—the others being in the Ganges River basin in the Indian subcontinent and between the Yellow and Yangtze rivers in present day China.

Over the next 1800 years, the disease remained in these population-dense areas, because smaller towns and hamlets could not sustain continual interhuman transmission. About 700 CE, with the rise of Islam in the Middle East and the spread of religion and trade across North Africa and south into sub-Saharan Africa, smallpox and other diseases took hold.

## COLONIZATION

**Figure 2.**
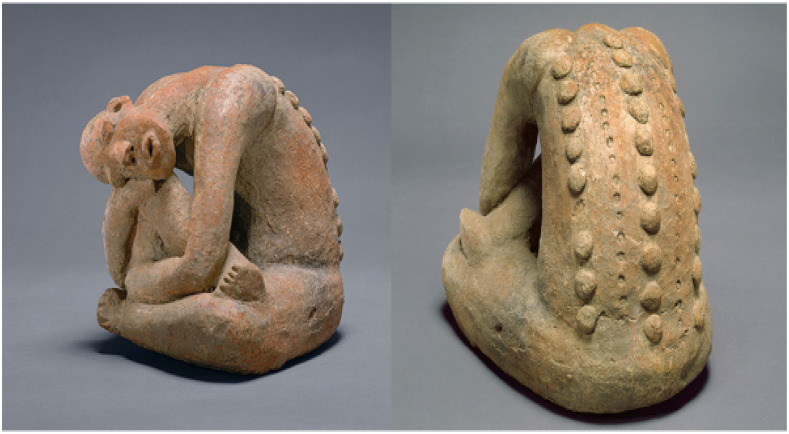
Smallpox in Art. Terra cotta statue, 13th century, Mali empire. Metropolitan Museum of Art, NYC. Photo provided by Kevin Tervala, Baltimore Museum of Art.

Over the next several hundred years, the great African kingdoms prospered. They fostered great art, architecture, and political stability. Figure 2 shows what may have been smallpox lesions from a statue in Mali dated thirteenth century (Figure 2).

By the late 1400s and well into the 1800s, the European powers arrived and established territorial extensions of their own kingdoms. First Portugal, then France, Belgium, Britain, Germany, Holland, and Spain. This influence lasts to this day.

## DISEASES AND DISEASE CONTROL

**Figure 3.**
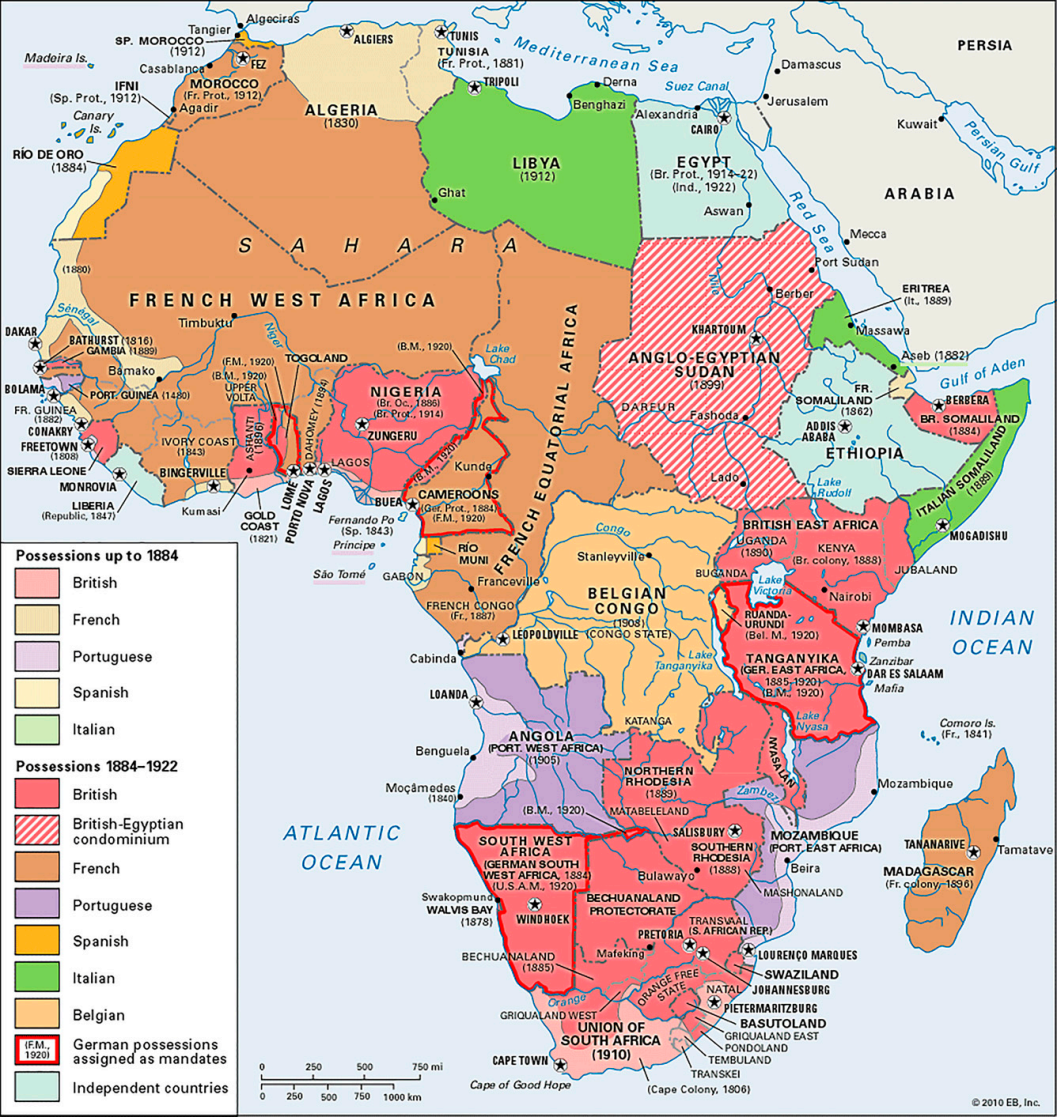
European colonial possessions in Africa, from 1600s to 1922. Figure provided by Kevin Tervala of Baltimore Museum of Art.

The colonizers were clear in their goals: to establish outposts for expediting their commercial interests. This included subjugating the locals—often by a divide and conquer strategy. Divisions of territory were made geographically in Europe, often without great attention to ethnic boundaries (Figure 3).

The expatriate communities concentrated along the coastlines in the capitals and in other large cities. These areas benefitted preferentially from public health measures which, beginning in the late 1800s, included vaccination against smallpox with partially effective liquid vaccines.

During the colonial period, the foreign military served as senior clinicians and public health supervisors. Civil servants and military officers trained in tropical diseases in Lisbon, Antwerp, London, Liverpool, Marseille, Bordeaux, and elsewhere provided the leadership and staffing of rural outposts. It was soon found that these isolated rural populations were afflicted with many medical conditions requiring attention.^4^

## SLEEPING SICKNESS IN THE CONGO BASIN AND OTHER AREAS OF SUB-SAHARAN AFRICA

The great campaign against sleeping sickness led by Eugene Jamot of France started in the Cameroons in the 1920s and slowly went north, village by village. Jamot trained illiterate microscopists to look for trypanosomes in the tissue taken from the nodules in the necks or cerebrospinal fluid of suspected patients with sleeping sickness.^5^ They also saw much smallpox.

Similar mobile teams were deployed in Anglophone West Africa focusing also on onchocerciasis, schistosomiasis, and leprosy while also giving smallpox vaccinations.^6^ Liquid smallpox vaccine was not heat stable. Vaccine success rates reflected by pustules on the arm were often less than 50%.^7,8^

Despite hundreds of thousands of smallpox vaccinations given in West and Central Africa—many more than the censused population numbers—periodic epidemics of smallpox raged in Africa. The real numbers were 10–100 times higher because of incomplete reporting.

## WHO PROGRAMS

In 1948, the WHO was formed with altruistic goals to develop health policies, establish disease surveillance systems, and promote control and elimination programs, of which malaria control relying on DDT for indoor house spraying was the most prominent activity.

## SMALLPOX ELIMINATION

In 1959, the Soviet delegation introduced a resolution for a smallpox eradication program to the World Health Assembly.^3^ For the next 8 years, little progress was made: few staff and funds came to the WHO for smallpox work. In 1967, an intensified eradication program began after another smallpox eradication resolution was endorsed by the assembly. About then, U.S. President Lyndon Johnson supported smallpox eradication efforts in West and Central Africa as part of the global program.^9^

The WHO requested that the director of the global smallpox program come from the United States—the country that had raised the resolution for an intensified program. D. A. Henderson of the CDC was chosen as the leader of the Smallpox Eradication Unit.^10^

## STATUS OF SMALLPOX—1967

**Figure 4.**
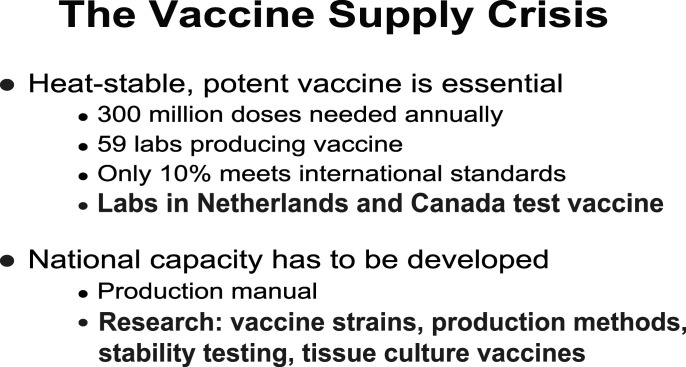
The vaccine supply crisis.

One of the first things done by the Smallpox Unit in Geneva was to look at the quality of vaccine being used by countries. It was found in 1967 that only 10% of the laboratories producing smallpox vaccines met standards for potency, stability, and purity (Figure 4). Two reference laboratories—Connaught in Canada and the Rijks Institute in Bilthoven, the Netherlands—tested products from 59 vaccine manufacturers.^3^

Seven countries of the 20 in the West and Central African program were among those with the highest incidence of smallpox in the world (Table 1).^11^

**Table 1:** Countries with the Highest Smallpox Rates, 1967

Country	Cases/100,000 population
**Sierra Leone**	**68.3**
**Guinea**	**40.2**
**Benin (Dahomey)**	**32.1**
**Niger**	**31.7**
**Togo**	**17.3**
India	15.3
Tanzania	13.4
Indonesia	11.7
Pakistan	10.4
**Congo (Brazzaville)**	**9.0**
**Nigeria**	**7.8**
Brazil	5.1

Foege et al.^11^ Bold = Countries in the West and Central African Smallpox Eradication-Measles Control Program.

Measles vaccine had been developed in the United States in the early 1960s at Merck, and the Minister of Health of Burkina Faso (then Upper Volta) visited the United States in the 1960s and invited the U.S. government to test feasibility of deployment of this vaccine in West Africa. After 2 years of field technology testing by CDC staff supported by the USAID, and country negotiation, the West and Central African smallpox–measles program was conceived and implemented. I lived in Guinea for 2 years and Burkina Faso for another 4 years during this program, and then in Geneva working on the final phases of eradication from 1977 to 1980.

## TECHNOLOGIES AND INNOVATIONS

Several scientific, technical, and managerial innovations led to the success of this program.

1. Freeze-dried vaccines: Both smallpox and measles vaccines were sent from the manufacturers to the field in heat stable, freeze-dried powder form along with diluent.^12^
2. Administration of vaccine: The Ped-O-Jet injector gun was used to inject the vaccine directly into the skin. An intradermal nuzzle was used for smallpox vaccine and a subcutaneous nozzle for measles.
3. Groups at risk: All persons were given smallpox vaccine as all were at risk. Children aged 6 months to 5 years were given measles vaccine. In Guinea, each team averaged 2,000 persons vaccinated a day per team over 2 years.^13,14^

Ped-O-Jet was effective when large numbers of persons could be assembled, as in Brazil and West and Central Africa. Toward the end of the West and Central African Program, the bifurcated needle was introduced into all of Africa and the endemic countries. This modification of a sewing machine needle allowed a drop of the vaccine to be caught between the prongs when dipped into the vaccine bottle. This allowed a great saving in vaccine. The procedure could be taught in a few minutes to virtually anyone, and the needles sterilized easily at night. Success rates were more than 95%.^3,14^

4. Strategy: The strategy when the West and Central African program began was to vaccinate at least 80% of the population—the original goal of the global program. It was believed that this level of coverage could result in herd immunity.

However, during the vaccine shortage in eastern Nigeria in 1967–1968 during the Biafran civil war, a CDC contract missionary, Bill Foege, former EIS officer, vaccinated only family members and close contacts of patients with smallpox. Other contacts at risk were vaccinated and put under surveillance. The cluster of outbreaks in eastern Nigeria completely melted. This approach became known as “selective ring vaccination” and “surveillance containment.”^15^

Emphasis was now put on aggressively finding every case of smallpox and stopping its spread. Epidemiological indices were used to find and contain cases—particularly seasonality. When the number of outbreaks and cases was lowest, during the rainy season, this was the best time to go to areas of high suspicion. Mass vaccination continued, but resources and publicity were now aimed toward surveillance and containment. An early track, trace, and cross-notification strategy was used. The last smallpox case was detected in Nigeria in May 1970.^11^

5. Diagnostic and research support: Supportive laboratory diagnosis and *Orthopoxvirus* research was built into the program from an early stage, first focusing on vaccine quality, but later on diagnostic conundrums.

In May 1971, a patient with a typical smallpox-like eruption was detected in the Democratic Republic of Congo, then Zaire, 10 months after the last smallpox case had occurred. WHO Poxvirus Collaborating laboratories in Moscow and Atlanta confirmed the diagnosis as monkeypox virus, an agent that had been the cause of outbreaks in animals in North American and European zoos. Over the next decade, several countries in West and Central Africa reported cases of human monkeypox. This newly detected disease in humans did not appear to transmit avidly from person to person.^16,17^ Smallpox vaccine protects against monkeypox, but immunity has waned since vaccination stopped in 1982 after eradication.^18^

Few longitudinal ecological studies of monkeypox have occurred. One rope squirrel with an eruption was found near a monkeypox outbreak, yet the natural history of this disease remains to be clarified.

Several recent large outbreaks of human monkeypox have been reported in Nigeria and DRC, as we heard at the smallpox symposium. We are now facing a decision on whether smallpox vaccine should again be used in areas where monkeypox is endemic.^19^

6. Operational innovations: The use of operations officers—managerial experts—was a major innovation in the West and Central African program. These persons were conversant with epidemiology and control, and became experts in logistics, communications, transport, personnel, and equipment—especially proper storage of vaccines. Management with surveillance was key to eradication.
7. Surveillance: Knowing what works and does not work in a timely, regular manner is now accepted by public health programs since smallpox. The WHO Weekly Epidemiological Record was used to transmit information on progress in each program.^10^ And the CDC had their own periodic summary for the West and Central African program, including progress in measles activities.
8. Field innovations: The best ideas throughout the program came from the field, not from headquarters.^3^ They were village-by-village searches for cases; feeding patients in their homes under guard; smallpox identification cards for searching in schools, villages, and camps; screening at festivals and the annual pilgrimage to Mecca; smallpox rumor registers; a monetary reward for reporting confirmed smallpox cases; detailed protocols for reporting and investigating cases; having petty cash funds in the hands of field-workers to hire local searchers and guards; moving staff from country to country when political winds changed, as occurred between Ethiopia and Somalia in the mid-1970s; and using Peace Corps workers as case detection agents and field supervisors.

## EXPANSION OF ELIMINATION

Whereas the West and Central African program was maturing, great progress was being made elsewhere. In the Americas, only Brazil and Argentina had disease. South America was the first continent to be certified smallpox-free by an independent commission. The major challenges remained with the Indian subcontinent and eastern Africa.

The big hurdle in India was convincing national and state health authorities that the surveillance containment strategy – including track, trace, isolate, vaccinate, report, and cross notify – was more effective than mass vaccination.

Village searches turned up thousands of outbreaks that had been unknown or unrecorded!

At one time, more than 150,000 field-workers in India alone were hunting smallpox. A detailed containment protocol for use at the local level was used. The last outbreak occurred in Ethiopia and then Somalia in 1976–1977, countries that were at war over land. Cases were occurring among nomads in the Ogaden desert, and moved into Somalia. Before containment occurred, more than 3,000 cases were identified and contained.^3^

The last naturally transmitted case occurred in Merca, coastal Somalia, on October 26, 1977. The last patients with naturally transmitted smallpox were Rahima Banu in Bangladesh, 1975, who had *Variola major*, and Ali Maaow Maalin, Somalia, 1977 with *V*. *minor*.

## SMALLPOX LESSONS FOR COVID CONTROL

There are many differences between COVID and smallpox. Coronaviruses are RNA viruses; as such they mutate, facilitating genetic and geographic tracking. A new mutant has been detected in England and South Africa and can be expected to spread worldwide. The variant is more transmissible between humans than the first isolates. This facilitates tracking the geographic spread. Seven coronaviruses are known to cause human disease. SARS-CoV-2 is believed to have come from an animal, perhaps as a bat virus transferred to another animal, and then with spillover to humans.^20^

Although there are many orthopoxviruses, variola, a DNA virus, is classified into major and minor. The major differences are in clinical and epidemiological presentation. There are public health lessons from smallpox that should be applied to public health control. The incubation period is 5–10 days for COVID-19 and 7–17 days for smallpox. Both are respiratory diseases transmitted mainly by large droplets. Important is the secondary attack rate among susceptibles.^21^

Considering the *R*_*o*_, for COVID-19, we are now seeing two–three, compared with three–six for smallpox and about 12 for measles.

There was no smallpox spread from asymptomatic persons to those in the incubation period. COVID does transmit during the incubation period.

Both conditions now have Food and Drug Administration (FDA)-approved treatments. During the program, we had no specific treatment for smallpox. With the specter of bioterrorism for smallpox, tecovirimat was recently approved by the FDA. Remdesivir is approved for COVID-19, but only shortens clinical illness, not mortality.

For smallpox, we had strong central leadership at the WHO, applying a public health model, with flexibility to change the model when new evidence accrued. We had a detailed operational plan. Each country adapted the plan based on local conditions; the new surveillance containment strategy using ring vaccination was central to the plan. High-quality vaccine, other supplies, and equipment usually came through a supply chain that was carefully managed centrally. Personnel from more than 70 countries worked as international civil servant leaders in endemic countries, serving the goals of the eradication program, rather than their own national agendas.^3^ Without this leadership and clarity, the program would have failed. The importance of international cooperation must be emphasized**.**

In closing, let me return to African innovations for COVID-19 control. Control measures, initiated by African countries, have muted the spread—particularly the rigorous implementation of case tracking, along with other mitigation measures. Nigeria, Uganda, Rwanda, and South Africa, among others, have developed highly successful methods in finding case contacts, testing them and taking measures to limit spread if they are positive. These countries had extensive experience establishing tracking systems for the Ebola outbreaks that have hit Africa since 1976—and also for polio, Lassa fever, Guinea worm, HIV/AIDS, and measles outbreaks.^23,24^

The innovations applied in Africa first resulted in the eradication of smallpox. Those being developed by African colleagues today will help us control COVID-19 and other perils now and in the future.

Thank you.
